# Implant Survival in Patients with Chronic Kidney Disease: A Case Report and Systematic Review of the Literature

**DOI:** 10.3390/ijerph20032401

**Published:** 2023-01-29

**Authors:** Iris Alla, Felice Lorusso, Sergio Alexandre Gehrke, Francesco Inchingolo, Maristella Di Carmine, Antonio Scarano

**Affiliations:** 1 Department of Innovative Technologies in Medicine and Dentistry, University of Chieti-Pescara, 66100 Chieti, Italy; 2 Department of Research, Bioface/PgO/UCAM, Montevideo 11100, Uruguay; 3 Department of Biotechnology, Universidad Católica de Murcia (UCAM), 30107 Murcia, Spain; 4 Department of Interdisciplinary Medicine, Section of Dentistry, University of Bari School of Medicine, Piazza G. Cesare, 11, 70124 Bari, Italy

**Keywords:** chronic kidney disease, dialysis, hemodialysis, renal failure, glomerulonephritis, hypophosphatemia, dental implant

## Abstract

Background: The aim of this systematic review and case reports was to evaluate osseointegration and implant survival rate in patients with chronic kidney disease. Methods: The paper screening process was conducted on electronic databases in order to identify clinical studies concerning the study topic. The literature data were evaluated for eligibility and studies were included for the qualitative synthesis. The case report concerned a male subject affected by renal disorders, a candidate for full arch immediate loading procedure. Results: The article screening process reported a total of 54 manuscripts and one paper identified through the manual search. At the end of the review process, a total of 45 articles were excluded while nine manuscripts were included for the descriptive synthesis. No significant complications or events were present during the intraoperative/post-operative phases. The clinical course reported no significant inflammation or symptoms. At follow-up, the rehabilitation was found to be functionally and aesthetically integrated with no complications, probing, or bone resorption. Conclusions: The available evidence supports the clinical efficacy of the early implant placement protocol. Present findings indicate that the early implant placement protocol results in implant outcomes similar to immediate and delayed placement protocols and a superior stability of peri-implant hard tissue compared with immediate implant placement.

## 1. Introduction

Implant therapy represents the treatment of choice for the rehabilitation of partial and total edentulous maxillary saddles at a level of long-term predictability [1]. This procedure is achieved by osseointegration of the implant fixture with the surrounding bone bases and by an ankylosis ratio at the bone-implant interface so that functional masticatory loads are supported [2,3,4]. This process develops according to a series of physiological events that take place from the stages of implant screw preparation [5] and placement, producing at that stage a stable relationship, also defined as primary stability, that is, by a mechanical–frictional relationship determined between the surfaces of the implant and the surrounding bone tissue [6,7]. Primary stability is influenced by multiple factors, including: bone density, contact surface, macro- and micro-geometry of the implant, surgical and implant site preparation technique [8,9,10,11]. This relationship of a mechanical nature is over time superseded by a subsequent stability of a biological nature, or secondary stability, determined instead by the neoformation of bone at the interface and coils of the implant that dynamically support its long-term maintenance and sustain functional loads [12,13]. Chronic kidney disease-mineral and bone disorder (CDK-MDB) is a bone disease (known as renal osteodystrophy) caused by deficits in bone mineralization and is a direct result of electrolyte and hormonal imbalance [14]. It is a complication of chronic renal failure that evolves into a systemic dysfunction of bone and mineral metabolism, including: biological serum abnormalities and mineral abnormalities, hyperphosphatemia, reduced serum vitamin D (VIT. D) levels, hypocalcemia, secondary hypoparathyroidism, increased epidermal growth factor-23 (EGF-23), abnormalities of bone growth and turnover, and vascular or soft tissue calcifications. CDK-MBD affects all aspects of bone physiology: bone volume, turnover, and bone mineralization, which increases the risk of bone fracture [15,16]. Bone remodeling is a lifelong process in which mature bone is replaced by remodeling into new bone tissue [17]. In adults, as remodeling proceeds, about 10% of bone tissue is replaced per year [13,14]. An imbalance in the regulation of either of the two subprocesses of bone remodeling results in CDK-MBD [14]. Assessment of bone turnover is the most important diagnostic means of monitoring the progression of CDK-MBD [14]. Patients with CDK-MBD may have normal, high, or low levels of bone turnover. Previous studies have shown that bone quality is significantly affected by CDK-MBD with variations at various levels of bone turnover [14,15]. So, changes in bone tissue with low turnover are detected by microstructural parameters, while changes in bone tissue with high turnover is manifested in changes in the quality of mineralization and in the nanomechanical properties of bone tissue [18]. These mechanisms include decreased mineral component in high turnover bone tissue, thinned trabeculae, and less spongy bone in low turnover bone tissue [18]. Subsequently, the jaw bones are affected by CDK-MBD, which can have major clinical complications for survival and osseointegration of the dental implant and success of regeneration therapy [18,19]. There is an increased risk of bone loss in patients with periodontitis and increased risk of bone fracture. However, according to the literature on bone, the latter behaves like other skeletal bones in CDK-MBD and there is currently no consensus on whether implant therapy is contraindicated in patients with CDK-MBD [18,19]. In an animal study of CDK-MBD on bone tissue quality, Allen et al. found important changes in bone quality during clinical observation of periodontal disease [16]. Zhao et al. evaluated residual alveolar volume in hemodialysis patients using CBTC computed tomography and found that the width and length of residual bone volume were significantly affected by the progression of CDK-MBD [20]. A similar study using CBTC for periodontal bone assessment reported significantly greater bone loss in patients with chronic renal failure compared with a healthy population [16]. However, Dijakewitz et al. reported that CDK-MBD seems to produce an alteration to the bone ridge volume, but the alveolar bone height and width were sufficient for implant placement in almost all subjects radiographically evaluated [21]. Frankenthal et al. found that even severe secondary hyperparathyroidism had no appreciable effect on the amount of alveolar bone [17]. An important limitation of these studies is the lack of assessment of bone density [18,19]. Using the same model, Zou et al. showed normal osseointegration of titanium implants, but the chronic kidney disease negatively affected implant osseointegration and primary stability in the early healing phase [18,19]. Bearing in mind that the process of bone turnover affects osteocyte density and causes alterations in osteocyte number and function, it is plausible to assume that osseointegration is affected by chronic kidney disease-mineral and bone disorder (CKD-MBD) [18,19]. The purpose of this study is to evaluate the response of chronic kidney disease patients undergoing implant pathology through a systematic literature review and case reports. In the second instance, the present review aimed to detect the influence of the loading protocol on dental implant treatment success.

## 2. Materials and Methods

### 2.1. Screening of Scientific Products

The literature search was conducted in accordance with the criteria of the PICO guidelines (population, intervention, comparison, outcome), as summarized in Table 1.

Systematic research data processing was carried out in accordance with the Preferred Reporting Items for Systematic Reviews and Meta-Analyses (PRISMA) guidelines [22].

The Boolean search was carried out according to the strategy described in Table 2 and conducted on electronic databases PubMed, EMBASE, and Google Scholar (27 September 2022).

### 2.2. Inclusion and Exclusion Criteria

For initial screening, the following criteria were applied: human clinical trials, prospective and retrospective studies, case series, and case reports with a minimum follow-up of 6 months after implant placement in English language without any restriction regarding the type of implant-prosthetic rehabilitation (e.g., fixed, mixed, total, partial), type of loading (e.g., immediate, early, delayed), and number of dental implants placed. Exclusion criteria were limited to systematic literature reviews, editorial letters, and in vitro studies. Then, after elimination of duplicates, full manuscript evaluation (full text) was performed for inclusion in the eligibility analysis.

### 2.3. Screening Process

Article screening and selection were carried out independently and blindly by two expert reviewers (FL and IA) for the purpose of evaluating the inclusion of scientific products in the descriptive analysis process. Duplicate and eliminated scientific articles were classified in each case by recording the reasons for exclusion in the review.

### 2.4. Data Analysis

The study data of included scientific products were recorded in a special database created using Excel software (Microsoft, Redmond, WA, USA) and classified according to the following characteristics: study design, population characteristics, number of patients treated/implants placed, analytical methodologies, inclusion/exclusion criteria, failed implants, follow-up, outcome.

## 3. Results

### 3.1. Preliminary Screening

**Figure 1:**
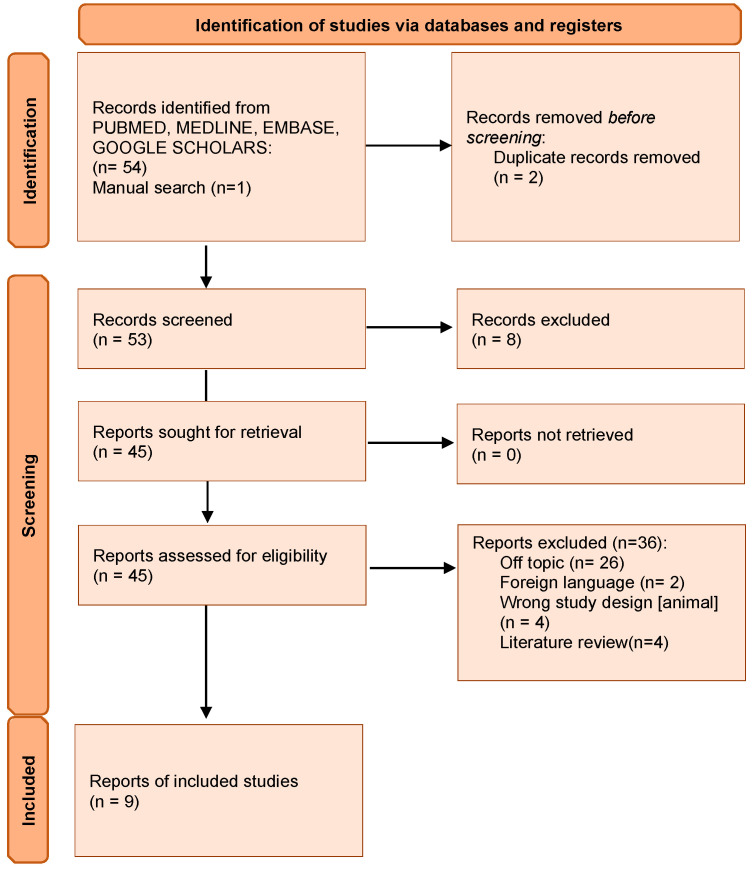
Preliminary Screening produced a total of 54 articles and one publication identified by manual screening.

The electronic database search phase (PubMed/Medline, EMBASE, Google Scholars) produced a total of 54 articles and one publication identified by manual screening. A total of two duplicates and eight products were removed from the preliminary evaluation phase. A total of 45 scientific products were submitted for eligibility evaluation. As a result, a total of 36 products were excluded from the synthesis process, including: 26 off-topic articles, two publications in non-compliant language, four scientific papers on animal model, and four literature reviews. A total of nine scientific papers were included in the descriptive analysis and synthesis (Figure 1).

### 3.2. Characteristics of the Studies Included

Therefore, the following were included in this systematic literature review: one retrospective case- control study [18], two prospective studies [16,18], one cohort study, two case series [18,19] and four case reports [21,23,24,25] (Table 2). Renal diseases associated with implant treatment were respectively: chronic renal failure with transplantation and immunosuppression therapy [26,27], chronic renal failure with long-term hemodialysis [23,28], hypophosphatemia and X-linked osteomalacia [29,30], Fraser syndrome [19], renal osteodystrophy [21], glomerulonephritis [25] (Table 3). The most frequently reported complication was implant failure in a total of three studies [26,28,30], while Lee et al. [29] reported implant hypermobility associated with discontinuation of therapies for treatment of X-linked hypophosphatemia [29]. In addition, bilateral retinal vein occlusion associated with implant placement procedure was associated in one case [25]. The follow-up period was very heterogeneous with a minimum of four months [31] and maximum of 34 years [27] (Table 3 and Table 4).

### 3.3. Case Report

**Figure 2:**
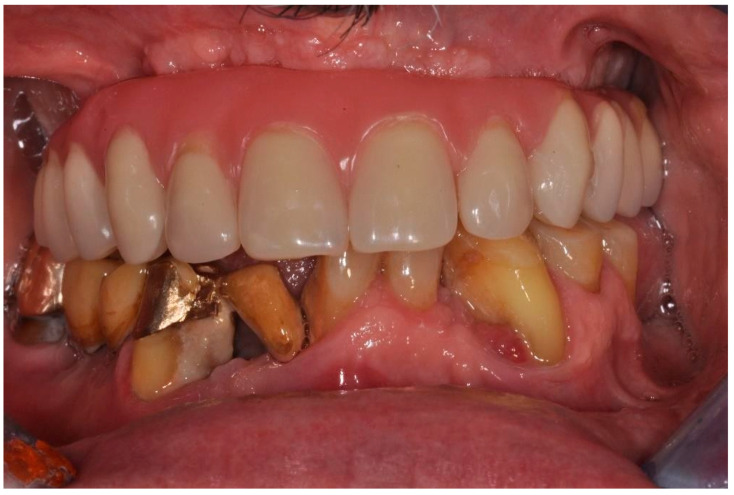
After jaw rehabilitation.

The study was conducted in accordance with the Declaration of Helsinki and informed consent was obtained from the patient. A male patient who was a candidate for upper and lower implant-prosthetic rehabilitation using fixed immediate-load prosthetics was treated at the Department of Innovative Technologies in Medicine and Dentistry. The subject was found to have chronic kidney disease undergoing treatment with hemodialysis (Figure 2).

**Figure 3:**
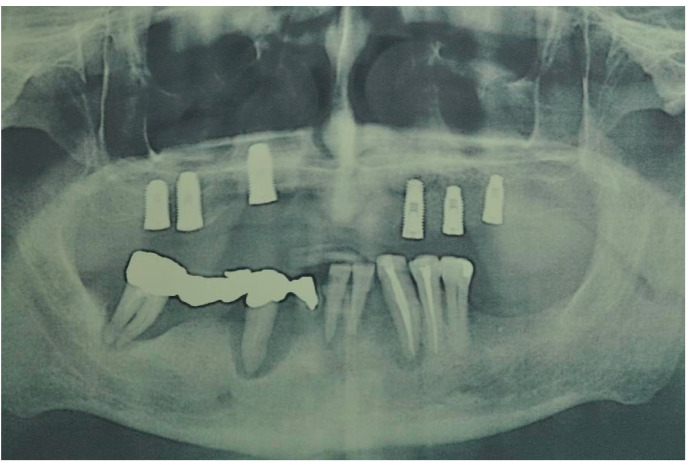
Postoperative orthopantomography in the upper arch.

The patient showed total edentulism of the upper arch with severe impairment of the mandibular dental elements. On OPT, aspects of bone atrophy as well as endo-parodontal lesions in elements 44 and 46 were visible (Figure 3).

Pre- and postoperative pharmacological supportive therapy included pharmacological premedication 1–3 days before (antibiotic-prophylaxis), local antisepsis, with chlorhexidine 0.12–0.2% mouthwash immediately before surgery (Better bacterial control if antisepsis is performed 2–3 days before surgery). Moreover, this is to be used in the postoperative period until suture removal. Local anesthesia was performed with vasoconstrictor (Articaine, Pierrel, Italy) 40 mg/mL, 1/100,000. Therefore, extractions of all compromised elements in the lower arch were performed: 47 44 41 and 31 33 34 35. (Extractions are performed in the most conservative manner so that the socket is ready to accommodate the implant. An ideal postextraction socket is one in which all corticals are intact. The blood clot will play a major role in supporting bone healing processes at the time of implant placement).

**Figure 4:**
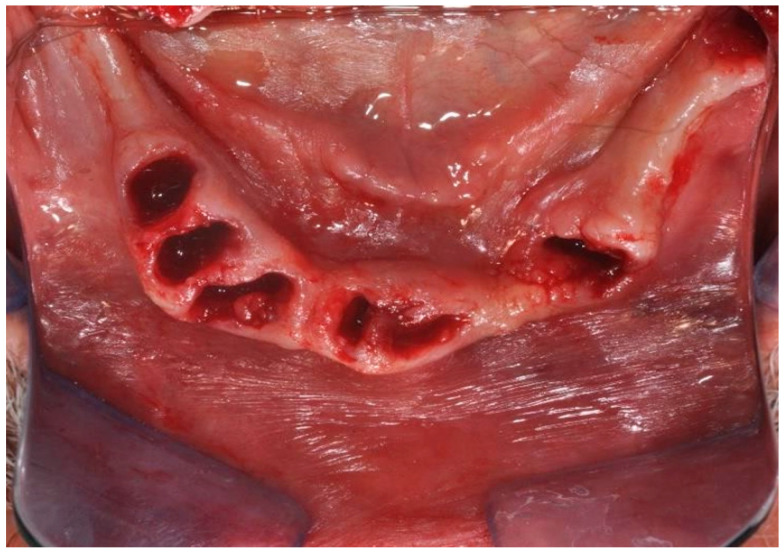
Surgical site after excision of compromised elements.

**Figure 5:**
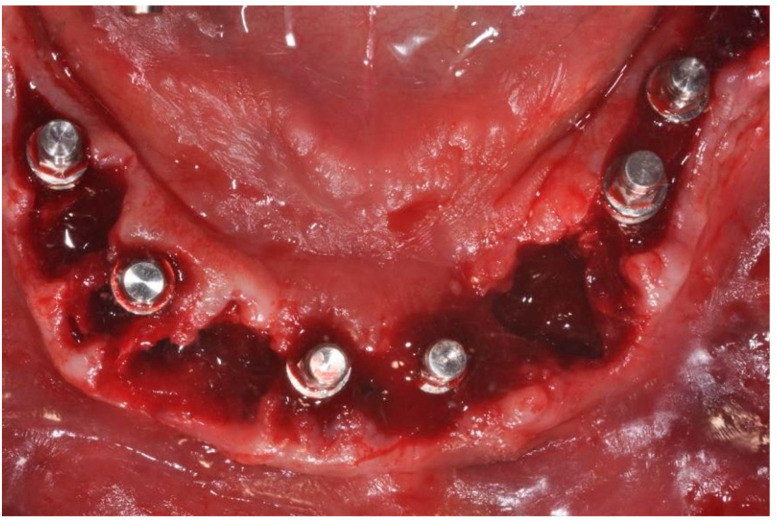
Surgical site after excision of compromised elements.

**Figure 6:**
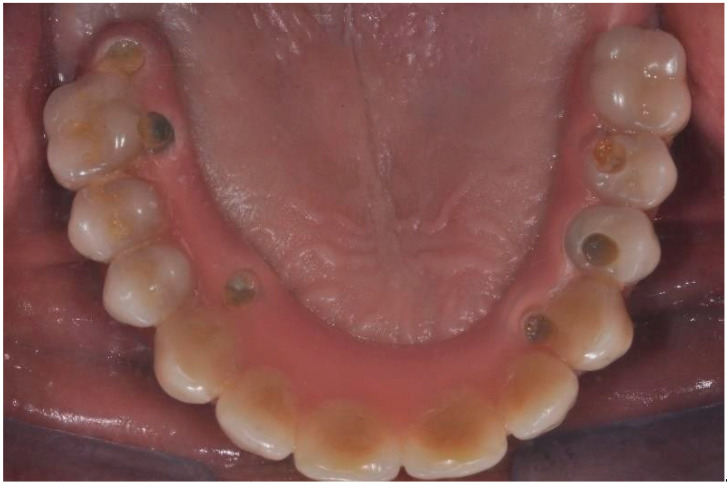
Occlusal view of the prosthetic rehabilitation.

**Figure 7:**
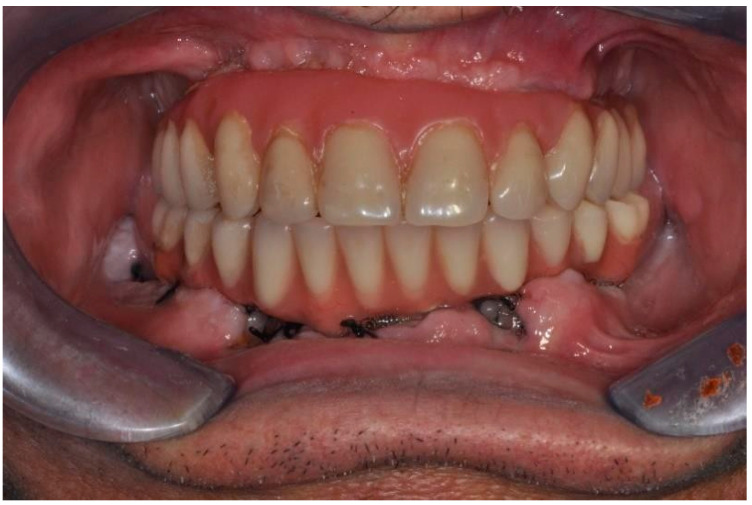
Frontal view of the prosthetic rehabilitation.

**Figure 8:**
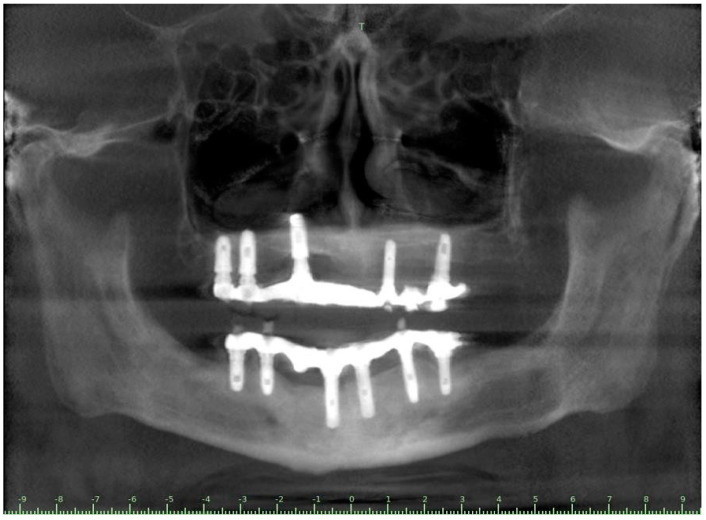
CBCT Panorex projection of the clinical case at the follow up.

**Figure 9:**
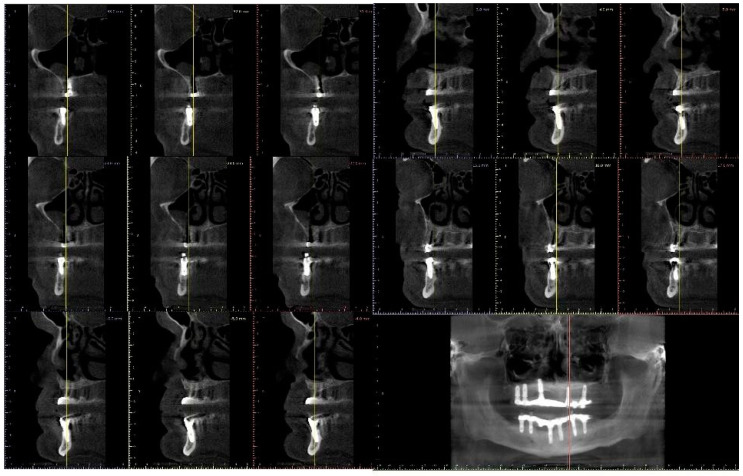
CBCT Cross section projection of the clinical case at the follow up.

A full-thickness ridge incision was then made with implant site preparation at 800 rpm 30 Ncm for the insertion of six implants (2P, Bone system, Milano, Italy) according to the drill sequence provided by the implant systematics (Figure 4, Figure 5, Figure 6, Figure 7, Figure 8 and Figure 9).

The temporary resin prosthesis was therefore placed according to the immediate loading protocol. Suturing of the access flap using 3/0 silk resorbable nonabsorbable thread was then placed, with follow-up at seven days and one year.

No significant complications or events were reported intraoperatively or in the postoperative course. At seven days, the surgical site appeared free of obvious phlogistic foci with virtually no symptoms. At follow-up, the rehabilitation was functionally and aesthetically well integrated in the absence of surgical-prosthetic complications. At follow-up, the peri-implant tissues had no pathological probing/foci of bone resorption.

## 4. Discussion

Kidney disease can produce a heterogeneous range of clinical conditions, differing in both type and severity, with a prevalence of around 5–10% of the general population, a percentage that is increasing given the strong causal association with two of the most prevalent diseases: hypertension and type II diabetes [27]. From a dental perspective and in outpatient clinical practice, the peri-operative and rehabilitative management of this category of patients may sometimes require a complex approach of a multidisciplinary nature [32]. The limit of the present investigation was represented by the very lack of clinical trials in favor of clinical reports/studies with lower level of evidence. In addition, no clinical reports included immediate loading protocols. In this way, the present investigation described a very rare clinical case where the patient was successfully rehabilitated with an immediate loaded implant prosthesis. As is evident from the present systematic review of the relevant literature, chronic kidney disease can present along a spectrum of different clinical forms, which differ in stages and degrees, with which protocols and specific therapies are associated that can produce significant effects on bone metabolism, thus playing a potentially central role in osseointegration processes in implant and regenerative therapy [33]. In addition, renal transplant recipients constitute a group of patients characterized by advanced stage of chronic disease and related complications, including renal osteodystrophy [18,23] and IgA nephropathy [23]. Osteodystrophy represents a condition of altered bone tissue metabolism that can potentially be a negative prognostic factor for implant treatment with a higher risk of failure [18,23]. In addition, this condition is associated with the effects of immunosuppressive therapy administered to the transplant patient, which has an often complex medical/drug protocol [26]. In addition, severe kidney disease can also sometimes be associated with congenital/hereditary factors as in the case of Fraser Syndrome, FAM20A mutation, or X chromosome-related hypophosphatemia [18,23].

### 4.1. Advanced Chronic Kidney Disease and IgA Nephropathy

IgA nephropathy is a kidney disease characterized by accumulation of type A immunoglobulins in the glomerulus, which is the fundamental unit of the kidney deputed to blood filtration and elimination of waste substances [23]. This condition is often associated with advanced chronic kidney disease, most often in patients who have already undergone hemodialysis with various comorbidities, and who, partly because of the latter, have a more pronounced deterioration of oral health and therefore require targeted dental care [23]. Flanagan et al., in this regard, performed a study in which implant treatment was evaluated in a patient with chronic kidney disease complicated by IgA nephropathy who was on standard treatment regimens of hemodialysis three times a week. Other comorbidities were associated with severe secondary hyperparathyroidism with concomitant intake of: calciomimetic agent, vit B supplement, amlodepine, besylate, metoprolol, paroxetine. The medical history also reported previous compound fractures of ulna and radius successfully treated surgically by hemotransfusion. In this regard, the authors report that mineral supplement therapy appears to be crucial, where serum calcium levels are important, especially for the prevention of inappropriate remodeling of the oral supportive bone tissue in these patients [23]. This is important expedient for the success of implant therapy, thanks to which even patients with systemic pictures characterized by advanced chronic kidney disease and complications on various levels can successfully undergo implant therapy.

### 4.2. Transplanted Patients

Patients who have received renal transplantation following a severe clinical picture of chronic kidney disease are patients undergoing immunosuppression drug therapy. Hernandez et al. in a study evaluated the efficacy of implant treatment in renal transplant recipients, and especially the long-term success of implant therapy, through a prospective controlled cohort study, showing excellent long-term results of implant therapy (over 98% survival rates) in immunosuppressed patients after renal transplantation. These patients had no more post-surgical complications and/or pain than case-controls of non-immunosuppressed patients, and after a long time, the incidence of peri-implantitis or bone loss in these patients was even lower than controls, although these differences were not significant. In the literature, no significant issues have been detected regarding the antibiotic prophylaxis, while studies included reported the administration of beta-lactam antibiotics (penicillins, cephalosporins), tetracycline, and macrolides with no correlated complications or events. Prudently, in subjects with severe chronic kidney disease with a potential accumulation risk, a dosage adjustment could be needed. The administration of non-steroidal anti-inflammatory drugs (NSAIDs) could also be affected by an accumulation risk. The analgesic therapy could be performed by paracetamol that does not require a dosage adjustment. Although the results were evaluated with a short follow-up period, when compared to other long-term reports of implant treatment outcomes using the same implant system in the general population, the results are comparable. Jemt et al. reported a survival rate of 90.9% after 15 years of follow-up in 76 patients and 450 implants, while Astrand et al. (2008) reported a survival over-rate of 99.2% after 20 years of follow-up in 21 patients and 123 implants [26]. These results demonstrate little or no impact of immunosuppression on healing and long-term outcome of implant therapy, although in the majority of patients in this study, implant therapy was performed in the so-called “late post- transplant period” with an average of 88 months between transplantation and implant surgery. This may be an important factor in assessing the period of initiation of implant treatment. Therefore, the late post-transplant period is preferred because in the immediate post-transplant period, the patient usually receives a high level of immunosuppression doses, which may lead to more side effects. Thus, this inevitably affects the healing of dental implants. Radzewsk et al. demonstrated implant success in a study in transplant patients undergoing treatment one year after renal transplantation. In the test group, they included patients without severe systemic disease, nonsmokers, with adequate bone health (appropriate bone volume and density), able to undergo implant therapy without the need to perform guided bone regeneration (GBR) [27]. By comparing the mean values of bone ridge level between the test group and the control group, this review showed that there were no significative differences between the two groups. From this, it follows that renal transplant patients can effectively undergo implant treatment while adhering to the required bone health conditions.

### 4.3. Renal Osteodystrophy

Renal osteodystrophy is a bone disorder secondary to chronic kidney disease that is established due to renal malfunction, which leads to altered blood calcium and phosphate levels and affects most dialysis patients. The resulting electrolyte and mineral imbalances, which systemically affect bone turnover, affect in particular cases the jaws by altering their bone quality, and this, in the treatment of an affected patient, requires proper diagnosis, case study, clinical, and radiographic evaluation. Dijakiewicz M. et al. [21] in a study evaluated implant success in patients with renal osteodystrophy by histological and histomorphometric analysis of jaw bone tissue samples and qualitative analysis of the bone mineral of the maxillary heads through EPR (electron paramagnetic resonance imaging), and finally an evaluation of biochemical markers of bone metabolism (calcium, phosphate, PTH, alkaline phosphatase). Analysis of the jaws showed that there was a decrease in the quantity and quality of bone tissue of the maxilla and mandible in renal osteodystrophy. However, according to recognized standards, these changes are not a contraindication to implant treatment. This is confirmed by clinical practice in which normal implant function was observed in a patient who had undergone implant therapy many years earlier, showing that there is no contraindication to implant treatment in this type of patients, and that these need more frequent professional counseling because of oral hygiene and microbiological control by RT-PCR. Only patients with inadequate status of the mandible were excluded from the study. In conclusion, the study shows the possibility of successful implant therapy in patients undergoing renal replacement therapy and presenting complication pictures, such as renal osteodystrophy, taking into account the specific circumstances of this category of patients at risk (immunosuppression, higher risk of infection, etc.) [21].

### 4.4. Enamel-Renal Syndrome (FAM 20A)

FAM 20A has recently been described as an enhancer of matrix protein phosphorylation within the secretory pathway. Dysfunction of FAM20A leads to a unique syndrome recognized as renal enamel syndrome or amelogenesis imperfecta with gingival fibromatosis. This is a very rare genetic malformation disorder characterized by hypoplastic amelogenesis imperfecta (hypoplasia of tooth enamel) and nephrocalcinosis (precipitation of calcium salts in kidney tissue). Mauprivez et al. reported in a study the case of implant treatment in a patient with enamel-renal syndrome. There were two symphysis implants to support an overdenture [28]. The first implant failed probably because of a very thin alveolar ridge. Given the first failed osseointegration of one implant, the team decided to perform a bone graft for the second implant, which was successful [28]. Therefore, given the appropriate preoperative analysis, implant success is possible in these patients.

### 4.5. X-Linked Hypophosphatemia

X-linked hypophosphatemia is a metabolic disease defined as X-linked congenital dominant osteomalacia. Affected individuals show a defect in the phosphate regulatory gene, resulting in increased urinary phosphate excretion, subsequent hypophosphatemia, and low vitamin D activity, involving lower levels of calcium, phosphate, and potassium. Clinical features are growth retardation, limb deformity, joint pain and evidence of osteoarthritis. Oral manifestations are: premature tooth loss due to formation of periapical abscesses, often without visible carious lesions; infections may be the result of bacterial infiltration through enamel infiltration or micro-cracks; pulpal chambers are enlarged, immature globular dentin. Patients need to replace lost teeth, so implant treatment is necessary. Ref. [29]. Radiographically, XLH is associated with deformities of the bone structure, such as a thin trabecular bone pattern and the total or partial absence of the lamina dura. From this, it follows that for implant therapy there may be alterations that can lead to failure and in any case the patient should be framed as a patient at risk. Various arrangements are therefore necessary that include monitoring of drug therapy, and an adequate healing period which, in these patients, given the slow bone turnover, it is necessary to prolong for the success of implant treatment [29]. In this regard, Lee et al. found in patients with XLH undergoing implant treatment, in particular in a 34-year-old woman, implant hypermobility. This condition was associated with the discontinuation of drug therapy to which the patient was subjected since the age of two years at which she was diagnosed with the disease. The discontinuation was carried out during the period of pregnancy and lactation antecedent to implant therapy. In another study by Frieberg et al., clinical evidence verified the possibility of performing implant treatment in patients with XLH, but prolonging the healing and waiting times for the prosthetic phase in which the implant is subjected to functional loading, all because of a slow bone response, a consequence of the pathology itself [29].

### 4.6. Fraser’s Syndrome

Fraser syndrome (FS) is a rare autosomal recessive malformation characterized by craniofacial dysmorphisms, cryptophthalmos, cutaneous syndactyly, and laryngeal abnormalities. Major and minor criteria are checked for diagnosis. Minor criteria include congenital malformations of the nose, ears or larynx, cleft lip/palate, skeletal defects, umbilical hernia, renal agenesis and mental retardation. Diagnosis was based on the presence of at least two major and one minor criteria or one major and four minor criteria. At the oral and dental level, patients with FS present with: facial asymmetry, an arched palate, hypodontia, microdontia, malocclusion, short roots and retained deciduous teeth [24]. Gallottini et al. in a study reported the case of a patient with FS whose medical history revealed that she had one kidney. The patient received implant treatment. Specifically, the therapy was performed at replacement and after extraction of maxillary central incisors for grade 3 mobility caused by very short roots. The alveolar bone tissue had low density, so manual osteotomes were used in combination with milling in the maxilla to improve initial implant stability. The implants were placed at the level of the bone, and the space between the alveolar bone and the dental implant was filled with xenogenic granular substitute with the aim of preserving the anatomy of the alveolar ridge. The implant of the right upper central incisor was installed with 15 Ncm and the implant of the left upper central incisor was installed with 20 Ncm [24]. After four months, the second stage of surgery was performed with reopening of the implant sites and installation of healing abutments. After six months of monthly follow- ups, the result of rehabilitation was excellent, both functionally and cosmetically. In addition, the patient’s individualized drug therapy included a weekly alendronate solution 70 mg (oral), calcium carbonate 600 mg daily tablets, eye lubricant drops, and hormone replacement therapy. For surgical treatment, the recommendation of the American Academy of Oral and Maxillofacial Surgery (AAOMS) was followed, and oral alendronate was discontinued for three months before and three months after surgery. Oral bisphosphonate use, although more rarely, has also been linked to osteonecrosis of the jaw. Moreover, in this particular case, the tooth roots of both teeth were very short and required less bone remodeling in the healing process. After four months, the successful osseointegration of the implants was evaluated. The surgeon noted low bone density at the time of installation and therefore opted for hand osteotomes in combination with milling in the maxilla to improve initial implant stability and subinstrumentation technique to achieve implant stability. The dental rehabilitation performed in this patient with FS improved masticatory function and provided aesthetic harmony to the patient’s face. FS is rare and dental and oral aspects have been scarcely explored to date. The report of this case suggests that care should be taken in patients with short-rooted FS and hypodontia. In addition, the placement of osseointegrated dental implants for rehabilitation in a syndromic patient and on bisphosphonate therapy is supported in this work.

### 4.7. PIP Treatment

Proton pump inhibitors are a class of drugs comprising molecules, such as omeprazole, lansoprazole, pantoprazole, esomeprazole, and rabeprazole, that act by reducing acid secretion at the gastric level. In a retrospective cohort study by Mauprivez et al., the aim was to investigate the association between proton pump inhibitor (PPI) intake and risk of dental implant failure. A total of 3559 implants were placed in 999 patients, with 178 implants reported as failures. Implant failure rates were 12.0% for PPI users and 4.5% for nonusers. Further, 3% of implants were lost. PPI intake has been shown to have a statistically significant negative value on implant survival rate. It follows that PPI intake may be associated with an increased risk of implant failure [21].

### 4.8. Glomerulonephritis

Maelescu et al. [25] in a study proposed the case of a patient with untreated Hepatitis C undergoing implant treatment (11 dental implants) who subsequently developed idiopathic glomerulonephritis with renal failure. Following implant treatment, in addition to glomerulonephritis, various systemic complications developed, including bilateral central retinal vein occlusion. Therefore, possible causes leading to the development of these complications were investigated. Dental procedures rarely induce ophthalmic complications. The injection of anesthetic solution into the oral cavity is the main factor in the development of these complications. There is no consensus on the exact etiology of ocular vascular complications after dental procedure. It is generally accepted that an anesthetic solution reaches the orbit through the vascular, neurological, or lymphatic network. However, most cases involve retinal artery occlusion by embolization or vasospasm, with no cases of retinal vein occlusion. There is a possibility that during many dental procedures the patient receives therapy with pro-coagulant substances for possible hemorrhage. The toxic effect of such drugs could also be found in some of the implanted materials and be a cause of vein occlusion, but the lack of knowledge about the toxic substances, irritants, and allergenic properties of dental materials prevents an exact conclusion on this topic [25]. The short-term prognosis for this case is moderate. The patient’s visual acuity is stable over time. The long-term prognosis, however, is uncertain. Due to a general imbalance caused by renal failure, the patient developed problems. Therefore, there is a risk of cerebrovascular and cardiovascular events. The patient could also develop diabetic retinopathy, because Tacrolimus, used as an immunosuppressant for renal transplantation, contributes to glucose intolerance, and chronic hepatitis C virus infection also appears to be a risk factor for the development of diabetes [25].

## 5. Conclusions

In conclusion, patients with kidney disease are placed in a broad category of patients at risk for implant procedures, likewise characterized by a wide heterogeneity of expression of pathologies. In this way, an accurate diagnosis, radiographic checks, and kidney function status are essential to avoid bone alterations and implant therapy risks. However, there are some insidious and critical phases: Firstly, during the implant placement phase, because of the low bone density, a condition by which patients with chronic kidney disease are often characterized. For this reason, in this phase, the surgical procedure may sometimes require very low speed preparation techniques: manual osteotomy. Secondly, another insidious phase is that of implant healing. Some authors agree to prolong these times even for about 9–12 months. Sometimes, this phase, therefore, may take longer. This needs to be coupled with a maintenance protocol that also takes into account adherence to pharmacological protocols, so that patients can be hormonally and mineral compensated, because these are patients that can present negative effects after time, e.g., with implant hypermobility, lead to the deterioration of implant health until failure. There are very few studies regarding the implant loading protocols that could be considered in the case of subjects with a fully stabilized chronic kidney function and a good clinical compliance.

## Figures and Tables

**Table 1:** Summary of the PICO (population, intervention, comparison, outcome) model.

Population\Patients	Intervention	Comparison	Outcomes
Subjects suffering from pathologies and disorders affecting the kidneys who are candidates for implant therapy.	Placement of dental implants in partial/total edentulous crests.	Patients who are healthy/not affected by kidney diseases and disorders	Implant efficacy and survival rate in patients with renal disorders.

**Table 2:** Screening strategy by Boolean search.

Search Strategies	
Keywords:	Advanced keyword search: (chronic kidney disease OR chronic renal disease OR dialysis OR hemodialysis OR renal failure OR glomerulonephritis OR hypophosphatemia) AND dental implant)
Databases	PubMed/Medline, EMBASE, Google Scholars

**Table 3:** Descriptive summary of included studies [Auth, Journal, Year, Methods, Population, Inc/excl criteria, Main outcome].

Authors	Journal	Year	Methods	Population	Inc./Excl. Criteria-Subject Characteristics	Main Outcome
Hernández G. et al. [26]	Clin Oral Impl Res	2019	Histology; CBCT	-28 partially edentulous RTP -28 control patients	Inc. criteria (1) Renal transplant (>18 months) immunosuppressive treatment. (2) Edentulism (>6 months). (3) Presence of teeth or fixed prosthesis in the opposing arch. (4) Sufficient bone volume for standard implant placement [no graft]. (5) Stable systemic health and clearance Excl. criteria: untreated periodontitis, smokers, radiotherapy, severe or uncontrolled metabolic diseases or lack of compliance.	-Similar ISR T and C groups (~98%) -PImuc [T:46.80%; C: 48.80%]. PImp [T:5.10% T;C: 8.10%]. -Similar wound healing and post-operative pain -No significant comorbidities (age, gender, oral diseases) (*p* > 0.05) -Significant difference of ISR for implants placed in the anterior mandible higher in C.
Flanagan D. et al. [23]	J Oral Implantol	2015	clinical and radiographic: full-mouth radiographic series, panoramic and a bimaxillary cone beam computerized scan and mounted study casts	-One patient	Subject of 34 yo male; severely carious teeth and associated chronic abscesses (kidney transplant delayed) Comorbidities: IgA nephropathy, tabagism, hypertension and secondary hyperparathyroidism. Hemodialysis three times weekly. Ph. Therapy: Nephrocaps vitamin (B) supplement, amlodepine, besylate, cinacalcet, metoprolol, paroxetine. Bilateral compound ulnar and radial fractures, 2 blood transfusions.	Successfully treated with dental implant-supported fixed prostheses: fixed bimaxillary porcelain fused to metal implant-supported complete dentures
Radzewsk et al. [27]	Implant Dentistry	2019	RVG	21 patients/24 implant 15 subjects/15 implants	Inc.T: 25 yo, 1 year after organ transplant, at least one tooth missing, suff. bone volume and density. [no graft]. Incl. C: No serious systemic diseases, >25 years of age, at least one tooth missing, bone volume and density. [no graft]. Excl. T/C: active periodontitis, occlusion disorders, bone and blood coagulation disorders, untreated dyslipidemia, or smoker.	T: 0.325 mm (min 0–max 0.95) C: 0.5 mm (min 0.15–max 1.8). CBL medians revealed no significant differences (*p* = 0.089).
Lee et al. [29]	Restorative Dentistry and Endodontics	2016	Rx, microscopic observ (Carl Zeiss Surgical GmbH)	case 1: 14 yo M (no implat th.) case 2: 38 yo F	XLH: X-linked hypophosphatemia	Implants with consistent hypermobility probably due to the interruption of medication (pregnancy and breastfeeding)
Gallottini et al. [24]	Spec Care Dentist	2018		One subject [16-yo Female]	FS: Fraser syndrome and kidney disease The patient presented four of the six major criteria for FS, (syndactyly, cryptophthalmos spectrum, one kidney agenesia, and tracheal anomaly). Several oral alterations, anodontia and short dental roots. Intervention: extractions and dental implant planning +administration of oral bisphosphonate (7 years) and osteopenia. -Osteopenia was attributed to the mildrenal insufficiency associated to one kidney and oestrogen deficiency	Total of 2 implants in the two maxillary central incisor sites with low bone density, hand osteotomes with drilling in the maxilla. Implant positioned with bone graft (Bio-Oss).
Mauprivez et al. [28]	The International Journal of Prosthodontics	2018	Panoramic radiograph, ceph., Tomography	Case 1: 18-yo M Case 2: (Retrospective cohort study) 3559 I. in 999 patients	Case 1: Enamel Renal Syndrome, FAM20A (family with sequence similarities 20A) gene mutations. Case 2: Medical the with PIP (CDK)	Case 1: 2 symphyseal I. to support an overdenture. One implant failure due to alveolar ridge thinness. Based on successful osseointegration of one implant, decided to perform a bone graft for 2 I. Case 2: PPI intake statistically significant negative effect for implant survival rate (hazard ratio 2.811; 95% CI: 1.139 to 6.937; *p* = 0.025)
Dijakiewicz M. et al. [21]	Oxford Journal	2007	-Radiometric analysis of panoramic tomography (DPT), -EPR (electron paramagnetic resonance), radiometric, densitometric, histological and histomorphometry implants simulation, biochemical markers of bone metabolism (calcium, phosphate, PTH, alkaline phosphatase).	T: 100 (HD) haemodialysis C: 50 healthy	ESRD (renal osteodystrophy)	-Decreased quantity and quality of bone tissue of the maxilla and mandible in renal osteodystrophy. -A total of 4 patients excluded due to the time of haemodialysis therapy (<bone density and bone decrease). -No exclusions on potential implant installation in the maxilla -normal function of the implants in patients suffering from renal osteodystrophy.
Friberg B. et al. [30]	International Journal of Periodontics & Restorative Dentistry	2013	Rx, Computed tomography scans	Case 1: 44 yo F Case 2: 43 yo M Case 3: 63 yo F	X-linked hypophosphatemia	Case 1: <periodontal bone support around remaining teeth, extend the healing period, bone response in relation to all 4 implants was unremarkable. Case 2: 4 regular-platform implants allowed to heal for 12 months prior to abutment connection. Fixed prosthesis positioned and normal bone response around all implants after 20 months post insertion. Case 3 overall majority of placed implants ended in early failure. Present outcome, with all 18 implants successfully in function after up to 5 years, better understanding of this slow bone turnover, allowing for markedly longer healing periods after extraction and implant placement
Malaescu M. et al. [25]	Romanian Journal of Ophtalmology	2019	-External and slit-lamp examination of the anterior segment of central retinal vein series of clinical, paraclinical and laboratory investigations -Blood count, coagulogram, and erythrocyte sedimentation rate;	-one subject: [54 yo, untreated chronic hepatitis C]	Bilateral central retinal vein occlusion after dental implant procedure. -high AST, ALT, and GGT levels due to hepatitis C infection and normal urea and creatinine values. Glucose levels were normal. -No significant findings at the cardio-vascular and rheumatologic exam. Nephrotic Syndrome and Stage III Renal Failure, leading to the diagnosis of Idiopathic Glomerulonephritis. Renal transplant bacterial endocarditis with MRSA and required minimally invasive mitral valve replacement	Decrease of visual acuity after the patient received 11 I., in over 1 year. Bilateral Central Retinal Vein Occlusion with Non-tractional Macular Edema OS > OD. In the following years, the general condition deteriorated, several systemic pathologies: arterial hypertension, hypertensive cardiomyopathy and nyha ii heart failure.

**Table 4:** Descriptive summary of included studies [Auth, Journal, Year, study findings, Implant failure, Follow up, Renal disease].

Authors	Journal	Year	Study Findings	Implant Failure	Follow Up	Renal Disease
Hernández G. et al. [26]	Clin Oral Impl Res	2019	AI, MCI and TP reduction in mineral density of the cortical and trabecular bone in CRF patients and more severely in patients under haemodialysis compared to peritoneal dialysis	1 IF in T	Mean follow-up of 116.8 months range from 84 to 192 months)	Renal transplant patients, are subjected pharmacological immunosuppression therapy
Flanagan D. et al. [23]	J Oral Implantol	2015	-Implant treatment for patients with IgA nephropathy (secondary hyperparathyroidism and osteodystrophy) may be successful. -Appropriate calcium therapy is important serum calcium to prevent inappropriate bone remodeling		2 years	Long-term dialysis patient with end-stage renal disease (ESRD)also referred to as chronic kidney disease (CKD) due to IgA nephropathy complicated by severe secondary hyperparathyroidism and renal osteodystrophy
Radzewsk et al. [27]	Implant Dentistry	2019	Patients with organ transplants can safely and effectively undergo dental implant treatment.		2 years	Renal transplant patients, are subjected to pharmacological immunosuppression therapy
Lee et al. [29]	Restorative Dentistry and Endodontics	2016	-Implant installation is not contraindicated for patients with XLH. -Uncertain possible prognoses. Interruption of medication may have negative influence on bone healing. -Blood calcium and phosphate concentrations in XLH patients should be monitored before surgery.	Case 2: hypermobility	Case 2: 1 year and 6 months	XLH: hereditary metabolic disease caused by the loss of phosphate through the renal tubules into the urine, and an associated decrease in serum calcium and potassium phosphate. XLH is the most common form of hypophosphatemic vitamin D-resistant rickets is a metabolic disorder involving <levels of Calcium and potassium phosphate due to the abnormal excretion of phosphate from the kidneys
Gallottini et al. [24]	Spec Care Dentist	2018	Imminent dental loss of the two upper central incisors (3 mobility-very short roots). -Suspended oral alendronate for 3 months before and 3 months after surgery. -Bisphosphonate oral use has also, albeit more rarely, been linked to osteonecrosis of the jaw; besides that, in this specific case, the dental roots of both teeth were very short, requiring less bone remodeling in the cicatricial process		4 months	FS, rare Ar malformation disorder
Mauprivez et al. [28]	The International Journal of Prosthodontics	2018	Case 1: Lost implant due to thinness of the alveolar ridge. Based on successful osseointegration of one implant, a bone graft for the second implant was performed. Case 2: Intake of Proton Pump Inhibitors-associated with the incidence of chronic kidney disease (Ckd), its progression or end-stage renal disease- is also associated with an increased risk of dental implant failure	Case 1: 1 IF Case 2: -45/178 IF implant failure rates 12.0% (30/250) for PPI users and 4.5% (148/3309) for nonusers. -45 /178 (25.3%) failed implants were lost up to abutment connection (6 in PPI users, 39 in nonusers), with an early-to-late failure ratio of 0.34:1.	Case 1: 2 (control for 1 st I.) - 3 (2 nd I.)-4 (control 2 nd) 6 months (control) Case 2: 34 years	Case 1:FAM20A (family with sequence similarities 20 A) Case 2: PIP associated with the incidence of chronic kidney disease (Ckd)
Dijakiewicz M. et al. [21]	Oxford Journal	2007	-No contraindication to implant treatment. -HD patients need more frequent professional advice on oral hygiene and microbiological control using RT-PCR		5 years	“Renal osteodystrophy, ESRD patients are immunodeficient, a condition caused by disturbances of the cellular and humoral immunological response”
Friberg B. et al. [30]	The International Journal of Periodontics & Restorative Dentistry	2013	Excellent marginal bone response seen over time; one could strongly recommend implant treatment in patients with XLH.	4 Implants failed (Case 3)	Case 1: 50 and 31 months. Case 2: 2 years. Other case study: 5 years.	XLH is an inborn X-linked D osteomalacia Patients show a defect in the phosphate-regulating gene with increased urinary excretion of phosphate, resulting in hypophosphatemia and low vitamin D activity
Malaescu M. et al. [25]	Romanian Journal of Ophtalmology	2019	Excluded acquired or genetic causes, Bilateral central retinal vein occlusion is a rare occurrence. The fact that the patient developed a drop in visual acuity in both eyes over a period of a few days pointed to the fact that systemic imbalance or disease must exist. -More severe complications like permanent amaurosis, orbital cellulitis, orbital abscess, and endophthalmitis were reported in the literature due to local anesthetic solution reaching the orbit through vascular, neurological, or lymphatic network. -The toxic effect of some of the implanted materials could also be a cause for the vein occlusion, due to hypotized toxic, irritant, and allergenic properties of dental materials.		4 years	Bilateral Central Retinal Vein Occlusion, Idiopathic Glomerulonephritis

## Data Availability

All experimental data to support the findings of this study are available contacting the corresponding author upon request.
